# An Assessment of GUCA1C Variants in Primary Congenital Glaucoma

**DOI:** 10.3390/genes12030359

**Published:** 2021-03-02

**Authors:** Emmanuelle Souzeau, Nicole Weisschuh, Jamie E. Craig, Francesca Pasutto, Karl-Wilhelm Koch

**Affiliations:** 1 Department of Ophthalmology, Flinders University, Flinders Medical Centre, Bedford Park, SA 5042, Australia; jamie.craig@flinders.edu.au; 2 Institute for Ophthalmic Research, Centre for Ophthalmology, University of Tübingen, 72076 Tübingen, Germany; nicole.weisschuh@uni-tuebingen.de; 3 Institute of Human Genetics, Friedrich-Alexander-Universität Erlangen-Nürnberg, 91054 Erlangen, Germany; Francesca.Pasutto@uk-erlangen.de; 4 Department of Neuroscience, Division of Biochemistry, University of Oldenburg, 26129 Oldenburg, Germany; karl.w.koch@uni-oldenburg.de; 5 Research Center for Neurosensory Sciences, University of Oldenburg, 26129 Oldenburg, Germany

**Keywords:** primary congenital glaucoma, GUCA1C, GCAP3

In the special issue “Molecular Genetics of Retinal Dystrophies”, Morales–Cámara and colleagues reported the association of a new candidate gene with primary congenital glaucoma (PCG) [1]. They identified a low-frequency nonsense homozygous variant (p.(Glu18Ter)) in the *GUCA1C* gene in two affected siblings with proposed autosomal recessive inheritance. Immunohistochemistry showed the presence of the encoded protein GCAP3 in human ocular tissues, including the ciliary epithelium and the retina. Additionally, the authors developed a *guca1c* knockout zebrafish and reported evidence of retinal ganglion cell apoptosis and the presence of GCAP3 in the same ocular tissues in adult wild-type zebrafish as in humans.

As previously reported by the ClinGen Gene Curation Working Group, the clinical validity of a gene–disease relationship can be assessed based on the strength of evidence that includes both genetic evidence (such as case-control studies or segregation data), and experimental evidence [2]. Here we aimed to investigate additional evidence regarding the reported association between *GUCA1C* variants and PCG.

In an effort to replicate these findings, we screened *GUCA1C* for variants in two separate cohorts of individuals with PCG, all predominantly of self-reported European ancestry [3,4]. This research was approved by the Southern Adelaide Clinical Human Research Ethics Committee (Adelaide, Australia) and the Institutional Review Board of the University of Erlangen–Nuremberg (Erlangen, Germany), and was conducted in accordance with the revised Declaration of Helsinki. All participants provided informed written consent. The Australian cohort was drawn from the Australian and New Zealand Registry of Advanced Glaucoma [3] while the European cohort was comprised of Italian and German individuals recruited at the Ophthalmology Department of the Erlangen University Clinic and the Genetic and Ophthalmology Unit of l’Azienda Socio-Sanitaria Territoriale Grande Ospedale Metropolitano Niguarda [4]. Exome sequencing was performed as previously described [4,5]. In brief, exomes were sequenced on a HiSeq 2000 (Illumina, San Diego, CA), following capture with the Agilent SureSelect version 4 system and on a SOLiD5500xl instrument (Life Technologies, Carlsbad, CA, USA). Reads were aligned to the hg19 reference using BWA, and duplicates were marked and removed with Picard. Variants were called using Sequence Alignment/Map tools and annotated using ANNOVAR, with allele frequencies from the Genome Aggregation Database (gnomAD, v2.1.1), and CADD scores. *GUCA1C* variants were annotated against the NP_005450.3 consensus sequence transcript. In the Australian cohorts (142 PCG patients and 238 healthy controls), we identified two patients and five control subjects heterozygous for the p.(Glu18Ter) variant, corresponding to allele frequencies of 0.007 and 0.011, respectively. In the German cohorts (38 PCG patients and 1389 healthy controls), p.(Glu18Ter) was not detected in any patients and was present in 15 controls, corresponding to allele frequencies of 0.0 and 0.011, respectively. We did not identify other variants predicted to be damaging in these individuals. A chi-square test indicated no significant difference in the overall allele frequency between patients (0.005) and controls (0.006) (p = 1.000). No individuals were homozygous for the variant and no other variants predicted to be damaging were identified in *GUCA1C* in our cases.

The p.(Glu18Ter) nonsense variant in *GUCA1C* reported by Morales–Cámara and colleagues has a low frequency in gnomAD [6]. In this database, the p.(Glu18Ter) variant is found on 1276/282,728 alleles (0.004513) (including in three homozygous individuals), with the highest allele frequency in non-Finnish Europeans (1053/129,060, 0.008159). The ACMG/AMP classification criteria for variant pathogenicity recommend that causative variants should not have an allelic frequency that is greater than expected for the disorder [7]. Following these guidelines, Whiffin and colleagues developed a statistical framework to calculate allele frequency cutoffs and assess whether variants may be too common to cause a Mendelian disease based on disease prevalence, genetic and allelic heterogeneity and penetrance for an autosomal recessive variant (https://www.cardiodb.org/allelefrequencyapp/ accessed date 8 June 2020) [8]. Using conservative estimates based on a prevalence of PCG in Europeans of 1/18,000 [9], a genetic heterogeneity of 20% (corresponding to the highest in Europeans for the *CYP1B1* gene) [10], no allelic heterogeneity and complete penetrance, the maximum credible population allele frequency for a pathogenic variant is 0.00333 and the maximum tolerated allele count in Europeans should not exceed 465. Therefore, the presence of 1053 alleles in non-Finnish Europeans in gnomAD v2.1.1 does not support pathogenicity of a fully penetrant *GUCA1C* p.(Glu18Ter) variant. Moreover, while there is little information on the phenotype of the three gnomAD subjects who are homozygous for this variant, it seems unlikely that all three of them suffered from PCG, since gnomAD excludes subjects known to have a severe childhood-onset disease (to which PCG belongs), as well as their first-degree relatives [6]. An alternative explanation for the observed frequencies, acknowledged by Morales–Cámara and colleagues, would be a pathogenic variant displaying incomplete penetrance and/or variable expressivity in a similar manner to some *CYP1B1* and *TEK* variants [11,12]. Using the same statistical framework, a penetrance of 15% would allow a maximum credible allelic frequency of 0.00861, which would be then compatible with the *GUCA1C* p.(Glu18Ter) allele frequency reported in gnomAD v2.1.1 in non-Finnish Europeans. Thus, further families identified with the *GUAC1C* p.(Glu18Ter) variant should be investigated with this in mind.

While the authors hypothesized that the p.(Glu18Ter) variant is likely to lead to a loss of function mechanism through mRNA degradation, a hypothesis supported by their finding of ganglion cell apoptosis in adult *guca1c* knockout zebrafish, there is another potential impact of the variant on the protein that should be discussed. The nonsense variant in *GUCA1C* identified by Morales–Cámara and colleagues occurs early in the transcript at codon 18 of 209 in exon 1 of 4 (consensus transcript ENST00000261047.3). While transcripts with premature termination codons occurring >50–55 nucleotides upstream from the last exon–exon junction are normally degraded by the cellular surveillance mechanism known as nonsense-mediated mRNA decay (NMD) [13], it has been shown that translation re-initiation can abrogate NMD in mammalian cells [14], since the ribosomes move forward to re-initiate translation at downstream start codons [15]. The nearest downstream internal start codons in *GUCA1C* are at codons 27 and 28. We performed an in silico prediction of the start codons using NetStart 1.0 (http://www.cbs.dtu.dk/services/NetStart/, accessed date 8 June 2020) [16] and found that the AUG at codon 28 is a probable translation start that has an even higher score (0.53) than the canonical start codon (0.43). Translation re-initiation has been shown to lead to hypomorphic or moderate clinical phenotypes in patients suffering from different inherited disorders [17,18,19,20,21]. How often translation re-initiation completely rescues the effect of nonsense variants is unknown, since these subjects would be asymptomatic. However, MacArthur and colleagues performed a systematic survey of loss-of-function variants in human protein-coding genes and estimated that human genomes on average contain ~100 high-confidence loss of function variants, with ~20 genes with homozygous or hemizygous loss of function variants [22]. They showed that most nonsense variants are found at the 3′- and 5′-ends, and explained this unequal distribution with a greater tolerance to truncation close to the end of the coding sequence and the rescue of truncated transcripts by transcriptional re-initiation at an alternative start codon. While functional evidence is needed to support a transcriptional re-initiation in the case of the *GUCA1C* p.(Glu18Ter) variant, this alternative predicted effect on the protein could support a role for p.(Glu18Ter) as a hypomorphic variant.

Finally, the mechanism of disease pathogenesis is important when assessing gene–disease relationships. Morales–Cámara and colleagues point to the regulation of intraocular pressure (IOP) through guanylate cyclases (GCs) and the fact that a GC-knockout mouse model developed a glaucoma-like phenotype based on studies from Ellis et al. (2011) and Buys et al. (2014) [23,24]. However, these studies investigated the soluble GC that is activated by nitric oxide (NO) and the membrane-bound GC that is regulated by natriuretic peptides (e.g., atrial natriuretic peptide (ANP)). The GC activators NO and ANP differ from guanylate cyclase-activating proteins (GCAPs) that are encoded by genes named *GUCA1A*, *GUCA1B* and *GUCA1C*. GCAPs activate photoreceptor cell-specific membrane-bound GCs in a calcium/magnesium-dependent manner [25]. *GUCA1C* encodes one of three GCAPs in the human retina (namely GCAP3), which shows an activation profile similar to other GCAP isoforms [26]. Furthermore, previous work have demonstrated that (a) the ANP-regulated hormone receptor GC is not regulated by GCAP [27] and that (b) the NO-soluble GC pathway is present in the outer retina, but is not located in photoreceptor outer segments. Outer segments of photoreceptor cells express membrane-bound GCAP-regulated GCs insensitive to NO [28]. Therefore, the GCs regulating IOP are distinct from the membrane-bound GCAP-regulated GCs of the human retina. Moreover, PCG is a developmental disorder characterised by abnormalities of the anterior chamber and the aqueous humor outflow structures of the eye. This is supported by the two most common genes causing PCG (*CYP1B1* and *TEK*) that are both associated with abnormalities of the trabecular meshwork and Schlemm’s canal [12,29]. Additional functional evidence assessing the molecular consequence of *GUCA1C* variants and evidence to support a role of the gene in the development of the anterior segment during embryogenesis are needed to support a gene–disease relationship with PCG.

In summary, the *GUCA1C* p.(Glu18Ter) variant reported by Morales–Cámara and colleagues has an allelic frequency in a population database that does not support pathogenicity under a highly penetrant autosomal recessive model, but would instead require a model of a hypomorphic variant or a null variant with incomplete penetrance and/or variable expressivity. Even under this model, *GUCA1C* p.(Glu18Ter) did not appear to be commonly associated with PCG in the cohort of Morales–Cámara and colleagues, or in our cohorts of 180 PCG exomes where we did not identify additional PCG cases homozygous for the *GUCA1C* p.(Glu18Ter) variant, or cases heterozygous for p.(Glu18Ter) with a second predicted damaging variant. The allele frequency of the p.(Glu18Ter) variant was also similar between our PCG cases and controls. Finally, predictive data and functional evidence are currently incomplete to support a gene–disease relationship. As acknowledged by Morales–Cámara and colleagues in their discussion, further studies are needed to establish whether *GUCA1C* variants are pathogenic in PCG.

## Data Availability

The data presented in this study are available on request from the corresponding author. The data are not publicly available due to ethical restrictions.
